# Who and How: Exploring the Preferred Senders and Channels of Mental Health Information for Wisconsin Farmers

**DOI:** 10.3390/ijerph16203836

**Published:** 2019-10-11

**Authors:** Josie M. Rudolphi, Richard Berg, Barbara Marlenga

**Affiliations:** 1 Department of Agricultural and Biological Engineering, University of Illinois, Urbana, IL 61801, USA; 2 Marshfield Clinic Research Institute, Marshfield, WI 54449, USA; berg.richard@marshfieldresearch.org (R.B.); barbaramarlenga@gmail.com (B.M.)

**Keywords:** farmer, mental health, occupational safety and health, agriculture

## Abstract

Unfavorable economic and environmental conditions have fueled the development of mental health resources and services for farmers. However, it is unclear who farmers want mental health information from (senders) and how they want mental health information delivered (channels). A self-administered questionnaire was used to determine the preferred senders of mental health information and the preferred channels of mental health information. Farmers were most receptive to receiving mental health information from medical providers, spouses/family members, and friends. Among the channels of information, respondents were interested in receiving mental health information from farm newspapers/magazines and one-on-one in person. Our findings have pragmatic implications for agricultural safety and health and public health organizations working to disseminate mental health information to farmers. Receptiveness to specific senders and channels of information among farmers should inform resource dispersion and future intervention.

## 1. Introduction

Agriculture has been recognized as a stressful industry [1]. Commonly reported stressors among farmers include commodity prices, time pressures, and environmental conditions [1,2,3,4]. There is converging evidence that the occupational stress of farming is associated with mental health problems, specifically anxiety and depression [2,5,6,7]. In response, commodity groups, National Institute of Occupational Safety and Health (NIOSH) agricultural safety and health centers, and farm organizations have developed a myriad of mental health resources. Some examples of these efforts include webinars on managing stress, fliers and brochures about mental health, and phone and text lines for farmers in crisis. In central Wisconsin, the community recognized the need for non-clinical intervention to promote farmers’ mental health and supported hosting Mental Health First Aid (MHFA) courses in three counties. However, it is unclear if the development and dissemination of mental health resources, such as webinars and text lines, were informed by farmers’ preference for mental health information. Similarly, while the MHFA curriculum has been thoroughly evaluated and standardized [8], there is no empirical guidance on who should be trained to deliver MHFA to farmers.

Communication theories provide guidance and a framework for effective and persuasive communication. The Sender-Message-Channel-Receiver (SMCR) model of communication is a form of linear communication. Simply, a sender delivers a message via a channel to the receiver [9]. In the model, the sender is the person or organization delivering a message or information [9]. The sender is charged with communicating effectively and should understand the culture, social norms, and expectations of the receiver to improve uptake of the message. The message is the content being delivered from the sender. The channel is the medium through which the message is delivered. Communication channels may be indirect, such as television or newspapers, or direct, such as face-to-face. Finally, the receiver is the individual who receives the message [9]. When considering delivery of mental health resources to farmers, the message (mental health information) and the receiver (farmers) are clearly defined. However, the sender of the message and the appropriate channels are in question.

Farmers in the Midwest have previously identified senders from whom they would be receptive to receiving agricultural safety and health information, which include Extension, insurance agents, and firefighters [10,11]. Similarly, farmers in the Midwest identified newspaper and magazine as trusted sources for agricultural safety and health information [11]. However, given the sensitivity and stigma surrounding mental health, it cannot be assumed, and we hypothesize, the preferred senders and channels of mental health information are the same as agricultural safety and health information. The primary objective of this descriptive study was to identify the people/organizations and channels from whom Midwest farmers would be receptive to receiving mental health information. A secondary objective was to compare preferred senders and channels of mental health information to the preferred senders and channels of agricultural safety and health information. Inquiring about senders and channels of mental health and agricultural safety and health information will allow for a contemporary, up-to-date assessment and comparison between the two among the same population for improved information and dissemination.

## 2. Materials and Methods

This cross-sectional, descriptive study was approved by the Institutional Review Board of Marshfield Clinic Health System.

### 2.1. Participants

Farmers were invited to participate in the study if they met the following inclusion criteria: (1) age 18 or over, (2) report farming as a full-time or part-time occupation, and (3) reported living in a three-county area.

### 2.2. Survey Delivery

Three hundred farmers were recruited from three Wisconsin counties (Clark, Marathon, and Wood). Participants were randomly selected from producer lists provided by UW-Extension in each of the three counties. Proportional random sampling was used based on the number of farms in each county in 2012 [12].

A paper-based survey was mailed to the 300 randomly selected farmers in central Wisconsin. Methods following Dillman for improved survey response were employed. Dillman’s method encourages personalized and repeated contact to encourage survey response [13]. Study participants received an initial mailing that included the survey, a postage paid return envelope, and $5. A reminder post card was mailed three weeks after the initial mailing. Three weeks after the post card was mailed a final request that included the survey and postage paid envelope was mailed to participants who had not responded. The study period began in January 2019.

### 2.3. Survey Instrument

A self-administered questionnaire was used to identify the senders and channels from which farmers would be receptive to receiving mental health information and agricultural safety and health information. The following sections were included in the questionnaire:

Definitions: Mental health information was defined as topics related to preventing and managing mental health disorders (i.e., anxiety, depression). Topics include, but are not limited to, stress management, common signs and symptoms of mental health disorders, and resources for managing mental health disorders. Agricultural safety and health information was defined as topics related to managing agricultural hazards and preventing agricultural-related injuries and illness. Topics include, but are not limited to, tractor/machinery safety, personal protective equipment, and livestock safety. Receptiveness was defined as you would be open to receiving mental health/agricultural safety and health information from the sender, comfortable discussing mental health/agricultural safety and health topics with the sender, and/or would seek out mental health/agricultural safety and health information from the sender.

Senders of Mental Health Information: Participants indicated their level of receptiveness to receiving mental health information from a list of 19 individuals (e.g., spouse, friends, and farmers) and organizations (e.g., commodity groups, religious groups). Response options ranged from 0 (not at all receptive) to 4 (extremely receptive). Participants were also asked to write in any senders they would be very or extremely receptive to receiving mental health information from but were not on the list of 19.

Channels of Mental Health Information: Participants indicated their level of interest in receiving mental health information from a list of 10 channels (e.g., in small group settings, in large group settings, and online). Response options ranged from 0 (not at all interested) to 3 (very interested).

Senders of Agricultural Safety and Health Information: Participants indicated their level of receptiveness to receiving agricultural health and safety information from a list of 19 individuals (e.g., spouse, friends, and farmers) and organizations (e.g., commodity groups, religious groups). Response options ranged from 0 (not at all receptive) to 4 (extremely receptive). Participants were also asked to write in any senders they would be very or extremely receptive to receiving agricultural safety and health information from but were not on the list of 19.

Channels of Agricultural Safety and Health: Participants indicated their level of interest in receiving agricultural safety and health information from a list of 10 channels (e.g., in small group settings, in large group settings, and online). Response options ranged from 0 (not at all interested) to 3 (very interested).

Personal Demographics: Participants responded to items inquiring about personal demographic characteristics including: age, race, gender, marital status, number of children, education level, alcohol and substance use, personal rating of physical health.

Farm Demographics: Participants responded to items inquiring about farm characteristics including: primary type of farm produce, secondary type of farm produce, number of people employed full-time on the farm, number of people employed part-time on the farm, primary role on the farm, number of family members working on the farm.

### 2.4. Data Analysis

Standard descriptive statistics are presented to summarize respondent characteristics and responses to the survey questions. Receptiveness to senders and channels of information on agricultural health and safety was compared to the corresponding responses for mental health by taking within-respondent differences and applying the Wilcoxon signed ranks test. For clarity in tables, adjacent categories in the 5-point (senders) and 4-point (channels) were combined into 3-point scales (not at all, somewhat, and very/extremely), but the statistical tests were computed using the full, original scales.

## 3. Results

Of the 300 surveys mailed, 159 farmers responded, and two surveys were undeliverable, resulting in a 53% response rate. The mean age of respondents was 56.1 years old (Table 1). Respondents were primarily male (89.9%) and white (93.1%). About a quarter reported working off the farm in some capacity. Commodities most commonly produced on farms included grain (64.2%), dairy cattle (62.9%) and forage (61.0%).

Respondents reported they would be most receptive to receiving mental health information from licensed medical providers (doctor), licensed mental health providers (psychologist), spouses/family members, and friends and least receptive to attorneys, agricultural bankers, and commodity groups. Similarly, farmers were most receptive to receiving agricultural safety and health information from spouses/family members, another farmer, friends, and licensed medical providers. Respondents were least receptive to receiving agricultural safety and health information from attorneys, agricultural bankers, and community groups (Table 2). Respondents did not write in any new or unique senders of mental health information or agricultural safety and health information.

When matching receptiveness to senders of agricultural safety and health and receptiveness to senders of mental health, 18 senders showed significant differences (Table 2). Respondents were much more receptive to receiving agricultural safety and health information than mental health information from 16 of the senders, with the two exceptions being licensed mental health providers (psychologist) and mental health organizations (National Association of Mental Illness).

Respondents were most interested in receiving mental health information and agricultural safety and health information from farm newspapers and magazines; 19.0% and 26.5% were very interested in this type of channel, respectively (Table 3). Over half of respondents were not at all interested in receiving mental health information from social media (71.4%) or one-on-one online (64.2%). Similarly, social media and one-on-one online were among the channels of least interest for agricultural safety and health information.

When matching interest in channels of agricultural safety and health information and interest in channels of mental health information, five channels showed significant differences (Table 3). For each of these five channels, respondents were much more interested in receiving agricultural safety and health information than mental health information.

## 4. Discussion

To our knowledge, this is the first study to identify senders from whom and channels through which Midwest farmers would be receptive to receiving MHFA and subsequent mental health information. Respondents were most receptive to receiving mental health information from medical providers, spouses/family members, and friends and least receptive to receiving mental health information from agribusiness personnel such as attorneys, agricultural bankers, and commodity groups. Respondents also identified spouses/family members, friends, and medical providers (doctor) as senders they would very/extremely receptive to receiving ASH information. However, farmers were also very/extremely receptive to receiving ASH information from agricultural health and safety specialists, firefighters/EMS, and Extension.

Among the channels of information, respondents were interested in receiving mental health and agricultural safety and health information from farm newspapers/magazines and one-on-one in person. Similarly, respondents were least interested in receiving mental health and agricultural safety and health information from the Internet, social media sites, and phone-lines.

While our sample was representative of the farming population in Wisconsin, primarily male, white, and middle-aged [12] we cannot be sure our sample is generalizable to the larger farming population of the United States. We searched the literature and compiled a list of 19 common senders of health and safety information. Additionally, we allowed room for respondents to identify senders not on our list. However, we cannot be sure we exhausted the list of the potential health and safety senders. As such, we may not have identified the ideal sender of mental health information. We also recognize available and preferred senders may differ geographically. Finally, we did not assess respondents’ interest in receiving mental health information. Individuals who were not at all interested in receiving mental health or agricultural safety and health information may report very low interest in the senders and channels and bias the findings of those respondents who would like to receive mental health and agricultural safety and health information.

To date, limited data exist showing from whom farmers would be receptive to receiving mental health information. However, data from rural populations are available. Similar to our findings, doctors and other health care providers have been identified as trusted sources for mental health information among rural women [14], for nutrition information among rural adults [15], and for breast cancer prevention information among rural women [16]. Interestingly, agribusiness personnel, such as agricultural bankers and sales people, though they have previously reported an interest in assisting their farmer clients [17] were not identified as a sender from whom respondents would be receptive to receiving mental health information or intervention. There were significant differences in the senders preferred by respondents for receiving mental health information versus agricultural safety and health information. Respondents appeared to be open to agricultural safety and health information from more senders than mental health information. For example, there were only two senders in which at least 40% of respondents said they would be very/extremely receptive to receiving mental health information: licensed medical providers (doctors) and licensed mental health providers (psychologists). Conversely, there were eight senders in which at least 40% of respondents said they would be very/extremely receptive to receiving ASH information. It appears there are more opportunities to deliver agricultural safety and health information when compared to mental health information. The lack of receptiveness should be explored to determine if this associated with stigma or general disinterest in the topic.

Respondents were most interested in receiving mental health information via farm newspapers/magazines and one-on-one, in person. Farm newspapers and magazines are popular sources of information among farmers for agricultural safety and health information [10,11], so our findings were not unexpected. However, while it was the highest rated channel, only a quarter of respondents reported they would be very interested in receiving mental health information from farm newspaper/magazines. Additionally, only 18.5% reported they would be very interested in receiving mental health information one-on-one, in person.

Much more obvious were the channels from which respondents were not interested in receiving mental health information, including social media (71.4%), one-on-one, online (64.3%), and the Internet (56.0%). Since the early 2000s, the Internet has been identified as a popular channel for information [18]. Increasingly, the Internet is becoming a trusted source of health information [19] including mental health information among rural women [20]. However, the Internet and social media were among the channels of least interest for mental health information among responders. While results of this study are important for mental health information dissemination, it also suggests lines of future research inquiry. Farmers identified friends and family as interpersonal sources from whom they would be receptive to receiving mental health information. However, a follow-up question is whether friends and family members feel confident and prepared to deliver mental health information to their farmer friends and family members. Furthermore, agribusiness personnel have expressed interest in promoting mental health among farmers, however, they were not identified as a preferred sender. Focus groups of farmers to explore what makes one sender more preferred than others would further inform mental health efforts.

It remains unclear how farmers want mental health information delivered. While preferred channels were identified, there were no overwhelming preferences. The lack of interest in receiving mental health information via the Internet, especially one-on-one, raises questions about the acceptability of tele-therapy among farmers. Mental health interventions delivered via telecommunication has been effective and well received among rural adults [21,22], however, the technology has not been evaluated among farmers. Focus groups with farmers to identify barriers and opportunities to various channels would be informative. For example, a focus group could determine if lack of interest in Internet resources is a function of connectivity in rural areas, distrust of the Internet, or lack of awareness of various mental health care delivery models.

## 5. Conclusions

Our findings have pragmatic implications for agricultural safety and health and public health organizations working to disseminate mental health information to farmers. Receptiveness to specific senders and channels of information among farmers should inform resource dissemination.

## Figures and Tables

**Table 1:** Demographic characteristics (*n* = 159).

Demographic Characteristics	*n* (%) or Mean (SD)
Age	56.1 (13.5)
Gender	
Male	149 (89.9)
Female	10 (10.1)
Race	
White	148 (93.1)
Black	0 (0.0)
Native American	1 (0.6)
Asian	0 (0.0)
Pacific Islander	0 (0.0)
Previous military experience	0 (0.0)
Highest level of education	
Up to high school diploma	71 (48.0)
Trade or some college	58 (39.2)
College graduate	19 (12.8)
Marital Status	
Single	20 (13.6)
Married	112 (76.2)
Divorced/separated	8 (5.4)
Widowed/widower	7 (4.8)
Off-farm employment status	
Full-time off-farm jobs	16 (11.2)
Part-time off-farm job	17 (11.9)
No off-farm job	110 (76.9)
Religious Status	
Protestant	34 (23.9)
Roman Catholic	66 (46.5)
Anabaptist	11 (7.7)
Other	17 (12.0)
No religion	14 (9.9)
Type of Production	
Conventional	125 (88.0)
Organic	6 (4.2)
Mixed	11 (7.7)
Farm’s 2017 gross sales	
< $10,000	13 (9.4)
$10,000–$49,999	28 (20.3)
$50,000–$99,999	22 (15.9)
> $100,000	62 (44.9)
Do not know	13 (9.4)
Commodities produced on farm	
Grain	102 (64.2)
Dairy	100 (62.9)
Forage	97 (61.0)
Beef	51 (32.1)
Poultry	8 (5.0)
Hogs	6 (3.8)
Produce	6 (3.8)
Cranberries	0 (0.0)
Other livestock	9 (5.7)
Other	11 (6.9)

Note: Not all categories sum to 100% due to missing data or opportunity to check more than one response option.

**Table 2:** Receptiveness of respondents to senders of mental health information (MH) and agricultural safety and health information (ASH) (*n* = 159).

Source	Not at All *n* (%)		Slightly/Moderately *n* (%)		Very/Extremely *n* (%)		Mean Difference ASH-MH ^a^
	MH	ASH	MH	ASH	MH	ASH	
Agricultural banker	65 (47.4)	56 (39.4)	64 (46.7)	70 (49.3)	8 (5.8)	16 (11.3)	0.28 *
Agricultural health and safety specialist	30 (21.6)	19 (13.8)	67 (48.2)	61 (44.2)	42 (30.2)	58 (42.0)	0.39 *
Attorney	86 (61.9)	66 (46.4)	45 (32.4)	61 (43.0)	8 (5.8)	15 (10.6)	0.37 *
Agricultural retailer (equipment, seed, etc.)	49 (35.3)	16 (11.3)	78 (56.5)	84 (59.6)	11 (8.0)	41 (29.1)	0.90 *
Another farmer, colleague	33 (23.7)	10 (7.0)	67 (48.2)	64 (44.8)	39 (28.1)	69 (48.3)	0.65 *
Extension agent	40 (28.8)	15 (10.6)	64 (46.0)	67 (47.5)	35 (25.2)	59 (41.8)	0.63 *
Firefighter/EMS	39 (28.5)	15 (10.6)	71 (51.8)	67 (47.5)	27 (19.7)	59 (41.8)	0.66 *
Spouse/family members	22 (15.6)	15 (10.5)	64 (45.4)	57 (39.9)	55 (39.0)	71 (49.7)	0.32 *
Friends	25 (17.7)	12 (8.3)	65 (46.1)	62 (43.1)	51 (36.2)	70 (48.6)	0.45 *
Neighbors	31 (22.1)	11 (7.7)	68 (48.6)	76 (53.5)	41 (29.3)	55 (38.7)	0.48 *
Insurance agent	67 (48.2)	31 (21.8)	62 (44.6)	89 (62.7)	10 (7.2)	22 (15.5)	0.57 *
Licensed medical provider (doctor)	21 (15.0)	15 (10.6)	56 (40.0)	62 (43.7)	63 (45.0)	65 (45.8)	0.05
Licensed mental health provider (psychologist)	31 (22.3)	33 (23.6)	45 (32.4)	65 (46.4)	63 (45.3)	42 (30.0)	−0.35 * (MH higher)
Professional agricultural association (Farm Bureau)	49 (35.0)	22 (15.4)	72 (51.4)	84 (58.7)	19 (13.6)	37 (25.9)	0.48 *
Veterinarian	40 (29.2)	18 (12.9)	77 (56.2)	63 (45.0)	20 (14.6)	59 (42.1)	0.78 *
Mental health organization (National Association of Mental Illness)	26 (18.7)	33 (23.6)	64 (46.0)	72 (51.4)	49 (35.3)	35 (25.0)	−0.32 *
Religious/Spiritual leaders (Pastor, priest)	33 (23.6)	32 (22.5)	65 (46.4)	77 (54.2)	42 (30.0)	33 (23.2)	−0.05 (MH higher)
Community group (Rotary)	58 (41.4)	43 (30.3)	71 (50.7)	85 (59.9)	11 (7.9)	14 (9.9)	0.22 *
Commodity group (Corn Gowers Association)	65 (47.1)	35 (24.8)	67 (48.6)	90 (63.8)	6 (4.3)	16 (11.3)	0.59 *

Note: Not all rows sum to 159 due to items skipped, ^a^ Wilcoxon Paired Sign Rank Test, original 5-point scale, mean of ASH receptiveness minus MH receptiveness. * Significant at <0.001.

**Table 3:** Interest of respondents in receiving mental health (MH) and agricultural safety and health (ASH) information from various channels (*n* = 159).

Channel	Not at all Interested *n* (%)		Somewhat/Moderately Interested *n* (%)		Very Interested *n* (%)		Mean Difference ASH-MH
	MH	ASH	MH	ASH	MH	ASH	
Television	61 (42.7)	41 (28.9)	67 (46.9)	88 (62.0)	15 (10.5)	13 (9.2)	0.24 *
Farm newspaper/magazine	27 (19.0)	16 (10.9)	88 (61.5)	92 (62.6)	27 (19.0)	39 (26.5)	0.38 *
Ag radio	47 (33.3)	29 (20.4)	80 (55.9)	98 (69.0)	14 (9.9)	15 (10.6)	0.35 *
Internet/websites	79 (56.0)	63 (44.1)	55 (38.5)	64 (44.8)	7 (5.0)	16 (11.2)	0.26 *
Social media (Facebook, etc.)	100 (71.4)	91 (64.5)	36 (25.2)	45 (31.9)	4 (2.9)	5 (3.5)	0.08
Phone line/hot line	74 (51.7)	72 (51.1)	58 (40.6)	63 (45.0)	11 (7.7)	6 (4.3)	−0.04
One-on-one, in person	45 (31.7)	30 (20.5)	76 (53.1)	89 (61.0)	21 (14.8)	27 (18.5)	0.22 *
One-on-one, online	90 (64.3)	93 (66.0)	46 (32.2)	47 (33.3)	4 (2.9)	1 (0.7)	0.00
Small group setting	52 (36.6)	45 (31.0)	73 (51.0)	81 (55.9)	17 (12.0)	19 (13.1)	0.06
Large group setting (conference, lecture)	62 (43.7)	55 (39.0)	69 (48.3)	75 (53.2)	11 (7.7)	11 (7.8)	0.05

Note: Not all rows sum to 159, a Wilcoxon Paired Sign Rank Test, original 5-point scale, mean ASH receptiveness minus mean MH receptiveness. * Significant at <0.001.

## References

[1] Booth N.J., Lloyd K. (2000). Stress in farmers. Int. J. Soc. Psychiatry.

[2] Furey E.M., Denis H., John M., Stephen K., Chris N. (2016). The roles of financial threat, social support., work stress, and mental distress in dairy farmers’ expectations of injury. Front Public Health.

[3] Walker J.L. (1988). Self-reported stress symptoms in farmers. J. Clin. Psychol..

[4] Austin E.K., Tonelle E.H., Anthony S.K., Jane L.R., Terry J.L., Hedda H.A., Sara S.A., David P., Brian J.K. (2018). Drought-related stress among farmers: Findings from the Australian Rural Mental Health Study. Med. J. Aust..

[5] Fraser C.E., Smith K.B., Judd F., Humphreys J.S., Fragar L.J., Henderson A. (2005). Farming and mental health problems and mental illness. Int. J. Soc. Psychiatry.

[6] Jones-Bitton A. (2019). Stress, anxiety, depression, and resilience in Canadian farmers. Soc. Psychiatry Psychiatr. Epidemiol..

[7] Onwuameze O.E., Paradiso S., Peek-Asa C., Donham K.J., Rautiainen R.H. (2013). Modifiable risk factors for depressed mood among farmers. Ann. Clin. Psychiatry.

[8] Kitchener B.A., Jorm A.F. (2002). Mental health first aid training for the public: Evaluation of effects on knowledge, attitudes and helping behavior. Psychiatry.

[9] Berlo D. (1960). The Process of Communication: An Introduction to Theory and Practice.

[10] Bendixsen C., Barnes K., Kieke B., Schenk D., Simich J., Keifer M. (2017). Sorting through the spheres of influence: Using modified pile sorting to describe who influences dairy farmers’ decision-making about safety. J. Agromedicine.

[11] Chiu S., Marsha C., Marizen R., Fred G. (2015). Where do agricultural producers get safety and health information?. J. Agromedicine.

[12] National Agricultural Statistics Service United States Department of Agriculture. https://www.nass.usda.gov/AgCensus/.

[13] Dillman D., Smyth J., Chrisian L. (2009). Mail and Internet Surveys: The Tailored Design Method.

[14] Historic Midwest Floods Sprout Worries About Mental Distress Among Farmers. https://www.huffpost.com/entry/farmers-mental-health-midwest-floods_b_5cc1c54ae4b09cae68a7b0e5.

[15] Zoellner J., Connell C., Bounds W., Crook L., Yadrick K. Nutrition Literacy Status and Preferred Nutrition Communication Channels Among Adults in the Lower Mississippi Delta. http://www.cdc.gov/pcd/issues/2009/oct/08_0016.htm.

[16] Kratzke C., Wilson S., Vilchis H. (2013). Reaching rural women: Breast cancer prevention information seeking behaviors and interest in Internet, cell phone, and text use. J. Community Health.

[17] Rudolphi J.M., Barnes K. (2019). Farmers’ Mental Health: Perceptions from Agribusiness Farm. Show Exhibitors. J Agromedicine..

[18] Internet/Broadband Fact. Sheet. https://www.pewinternet.org/fact-sheet/internet-broadband/.

[19] Hesse B.W., Nelson D.E., Kreps G.L., Croyle R.T., Arora N.K., Rimer B.K., Viswanath K. (2005). Trust and sources of health information-The impact of the Internet and its implications for health care providers: Findings from the first Health Information National Trends Survey. Arch. Int. Med..

[20] Simmons L.A., Qi S.W., Nancy Y., Heather M.B., Leslie J.C. (2015). Sources of health information among rural women in Western Kentucky. Public Health Nurs..

[21] Grubaugh A.L., Cain G.D., Elhai J.D., Patrick S.L., Frueh B.C. (2008). Attitudes toward medical and mental health care delivered via telehealth applications among rural and urban. Primary care patients. J. Nerv. Ment. Dis..

[22] Openshaw D.K., Jenny M., David L., Dan M., Christopher J., Susan T. (2012). Examining the satisfaction of women residing in rural Utah who received therapy for depression through teletherapy. J. Rural Ment. Health.

